# Supplementary material for: Surprising regulatory plasticity for the conserved HOG pathway in diverse Saccharomyces cerevisiae strains
Source: bioRxiv. 2025 Dec 28:2025.12.28.696518. Preprint. [Version 1] doi: 10.64898/2025.12.28.696518 (PMC12776145; doi:10.64898/2025.12.28.696518)
Supplement: 2 [file NIHPP2025.12.28.696518v1-supplement-2.pdf]

bioRxiv preprint doi: https://doi.org/10.64898/2025.12.28.696518; this version posted December 28, 2025. The copyright holder for this preprint (which was not certified by peer review) is the author/funder, who has granted bioRxiv a license to display the preprint in perpetuity. It is made available under aCC-BY 4.0 International license.

Supplemental Figures

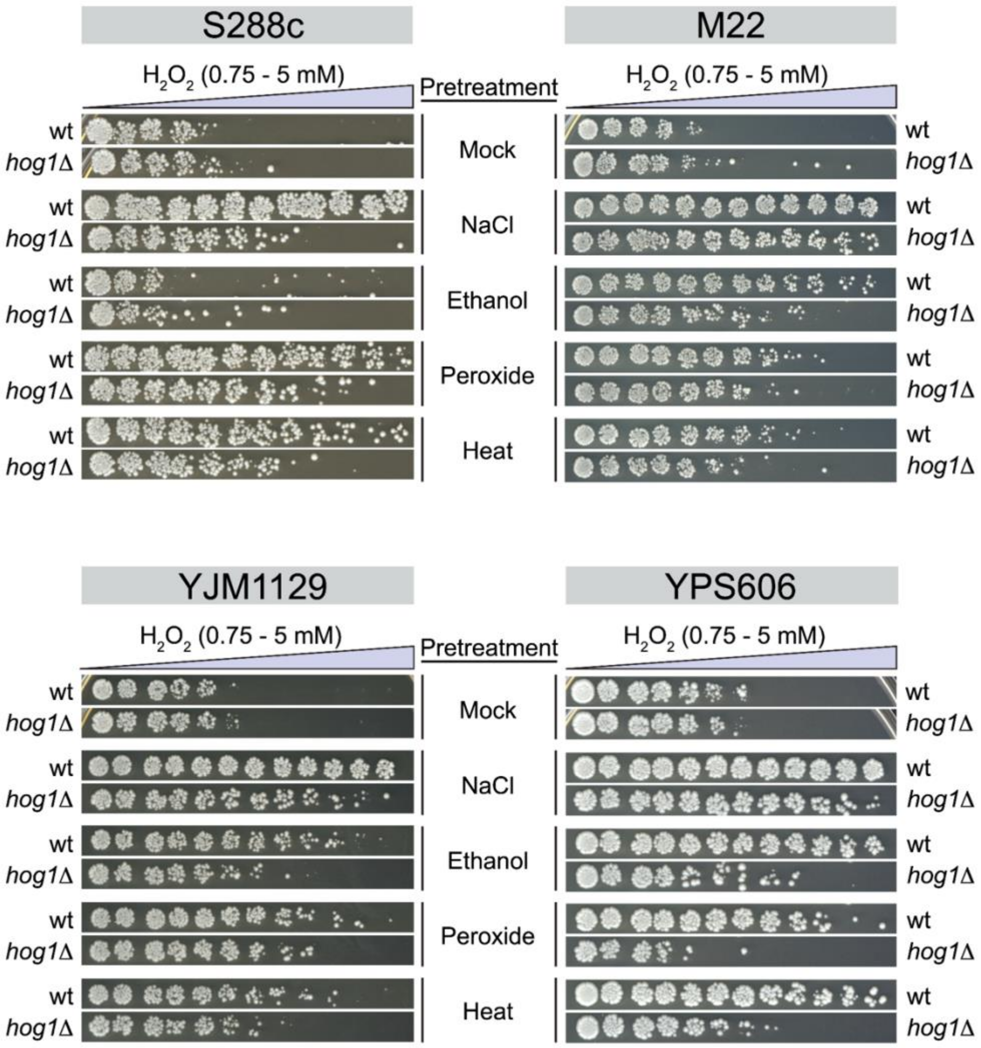

Figure S1. Representative H_2O_2 cross protection assays for all strains and conditions depicted in Figure 1.

bioRxiv preprint doi: https://doi.org/10.64898/2025.12.28.696518; this version posted December 28, 2025. The copyright holder for this preprint (which was not certified by peer review) is the author/funder, who has granted bioRxiv a license to display the preprint in perpetuity. It is made available under aCC-BY 4.0 International license.

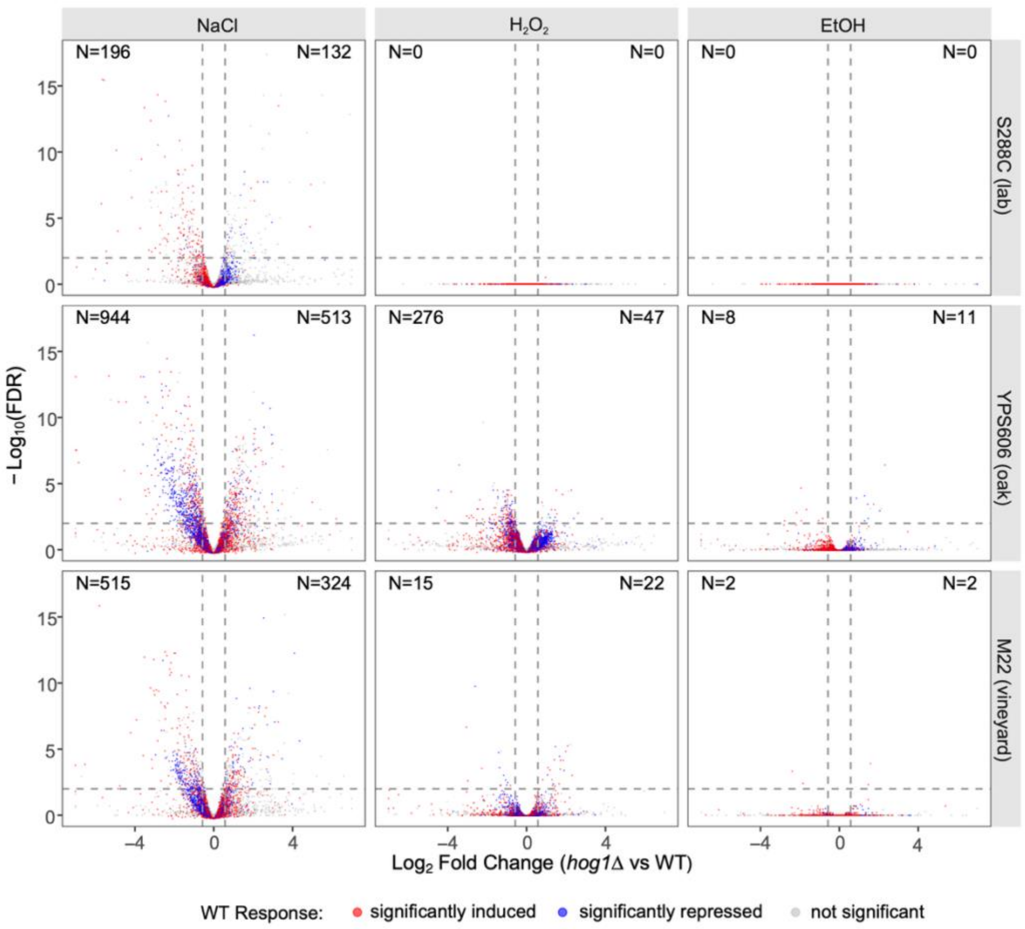

Figure S2. Hog1-dependent differential expression during non-osmotic stresses in wild strains is robust to subsampling and thus not due to differences in statistical power. Subsampling was performed by reducing the number of samples in wild strains to 3 (equal to S288C), and reads were subsampled down to the sample with lowest read counts. Differential expression analysis was performed in edgeR using the exact same parameters as for the full dataset. Volcano plots were performed as in Figure 2.

bioRxiv preprint doi: https://doi.org/10.64898/2025.12.28.696518; this version posted December 28, 2025. The copyright holder for this preprint (which was not certified by peer review) is the author/funder, who has granted bioRxiv a license to display the preprint in perpetuity. It is made available under aCC-BY 4.0 International license.

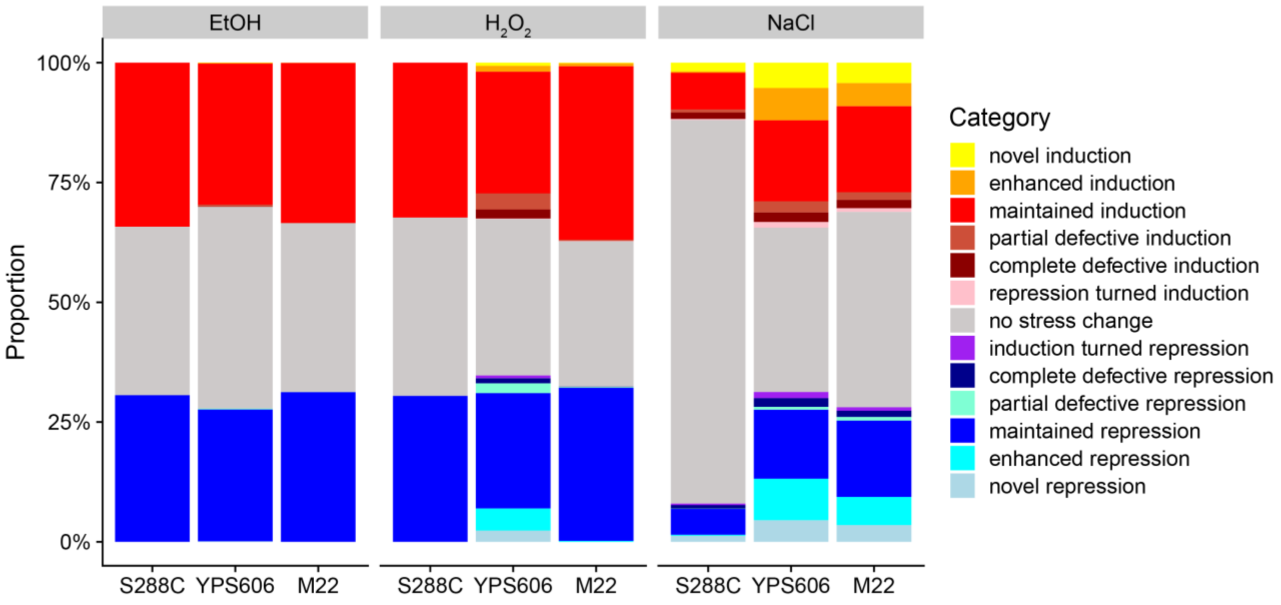

Figure S3. Strain-specific variation in classes of Hog1-regulated genes. The bars depict proportions of each class of genes. Genes unaffected by Hog1 fall into three major classes: no change in expression during stress (grey), induced during stress but unaffected by lack of Hog1(red), and repressed during stress but unaffected by lack of Hog1 (blue). All other classifications for Hog1-regulated genes are noted in the key.

bioRxiv preprint doi: https://doi.org/10.64898/2025.12.28.696518; this version posted December 28, 2025. The copyright holder for this preprint (which was not certified by peer review) is the author/funder, who has granted bioRxiv a license to display the preprint in perpetuity. It is made available under aCC-BY 4.0 International license.

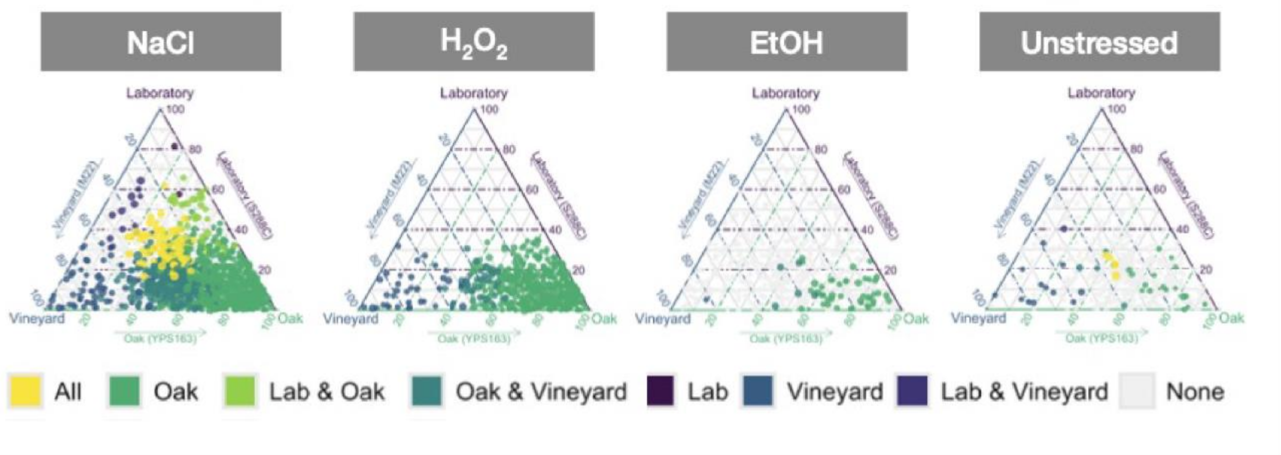

Figure S4. Shared and strain-specific Hog1 dependency for osmotic and non-osmotic stresses. Ternary plots show the relative magnitude of Hog1-dependent gene expression changes across all three strains as per Figure 3.

bioRxiv preprint doi: https://doi.org/10.64898/2025.12.28.696518; this version posted December 28, 2025. The copyright holder for this preprint (which was not certified by peer review) is the author/funder, who has granted bioRxiv a license to display the preprint in perpetuity. It is made available under aCC-BY 4.0 International license.

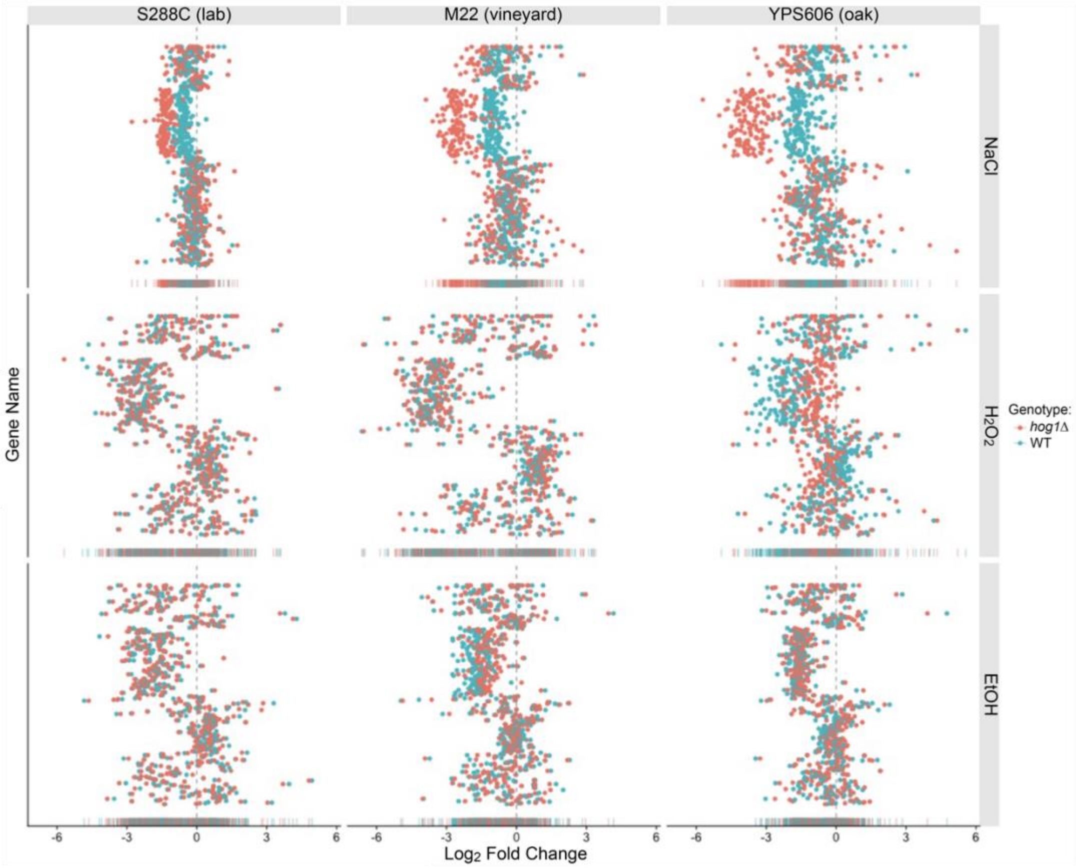

Figure S5. Hog1 specifically exhibits opposing effects on ribosomal protein (RP) gene regulation. All transcripts annotated as belonging to the GO term translation were sorted by gene name, which largely sorts the major classes of RP genes and ribosome biogenesis genes (Ribi).

bioRxiv preprint doi: https://doi.org/10.64898/2025.12.28.696518; this version posted December 28, 2025. The copyright holder for this preprint (which was not certified by peer review) is the author/funder, who has granted bioRxiv a license to display the preprint in perpetuity. It is made available under aCC-BY 4.0 International license.

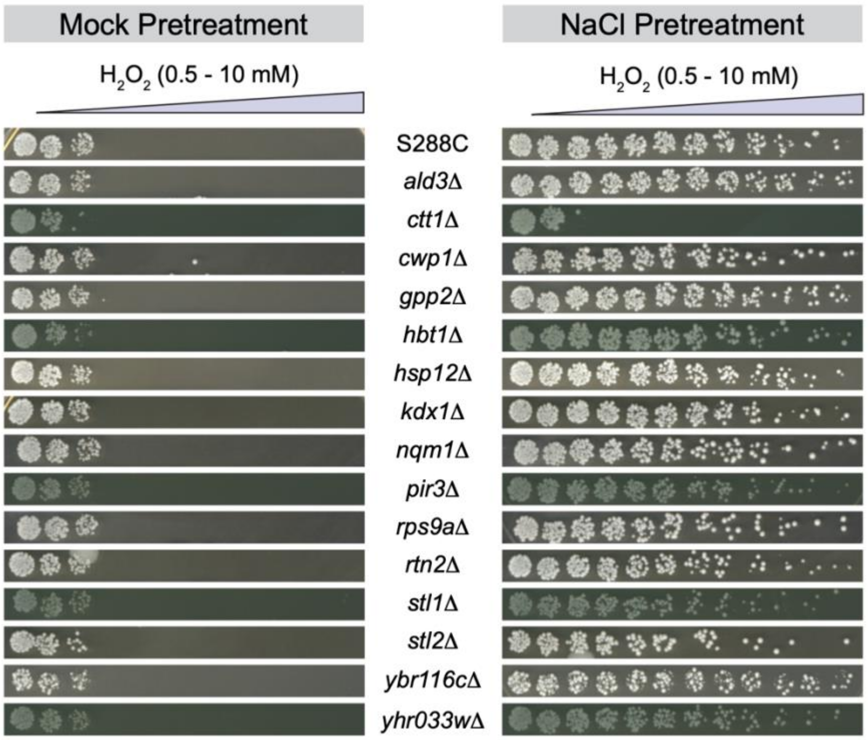

Figure S6. NaCl-induced cross protection phenotypes for mutants in genes implicated by correlation analysis. Strains from the YKO collection (S288C background) were chosen based on high expression-phenotype (MIC*) correlation.

bioRxiv preprint doi: https://doi.org/10.64898/2025.12.28.696518; this version posted December 28, 2025. The copyright holder for this preprint (which was not certified by peer review) is the author/funder, who has granted bioRxiv a license to display the preprint in perpetuity. It is made available under aCC-BY 4.0 International license.

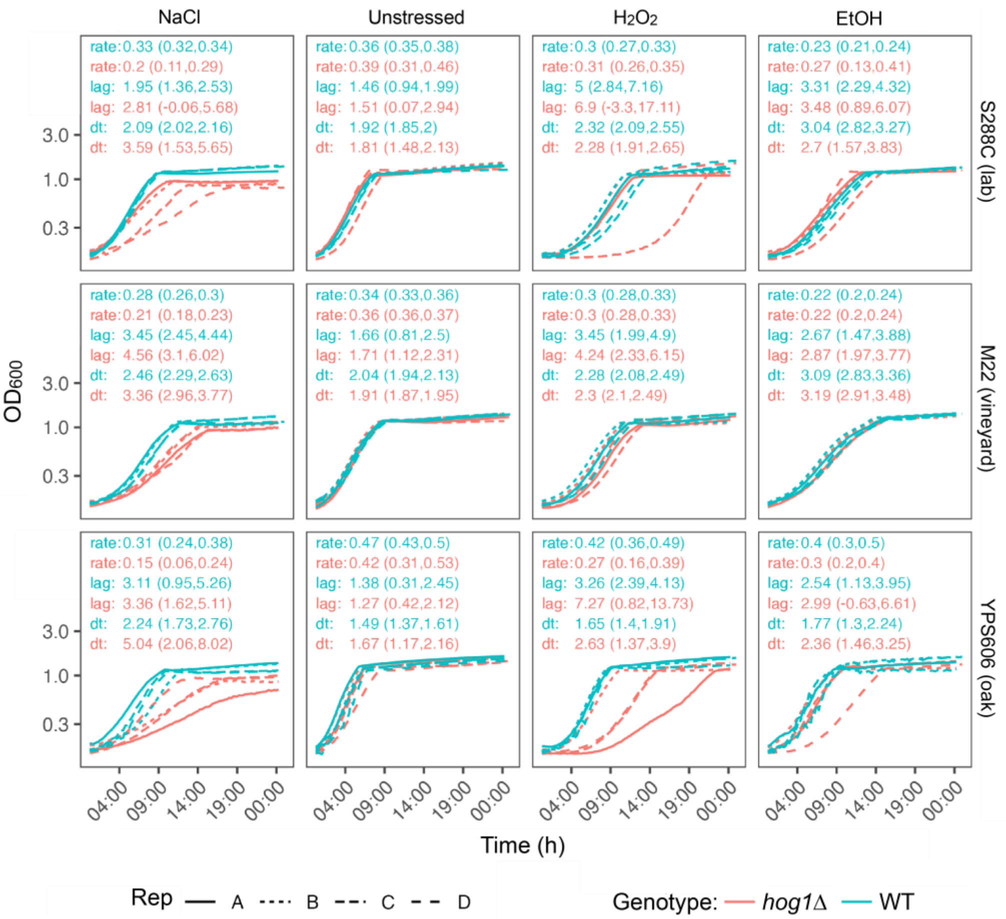

Figure S7. Growth analysis of wild-type and *hog1*Δ mutants under stress. Insets show maximum specific growth rates, lag time estimates, and doubling times (dt).

bioRxiv preprint doi: https://doi.org/10.64898/2025.12.28.696518; this version posted December 28, 2025. The copyright holder for this preprint (which was not certified by peer review) is the author/funder, who has granted bioRxiv a license to display the preprint in perpetuity. It is made available under aCC-BY 4.0 International license.

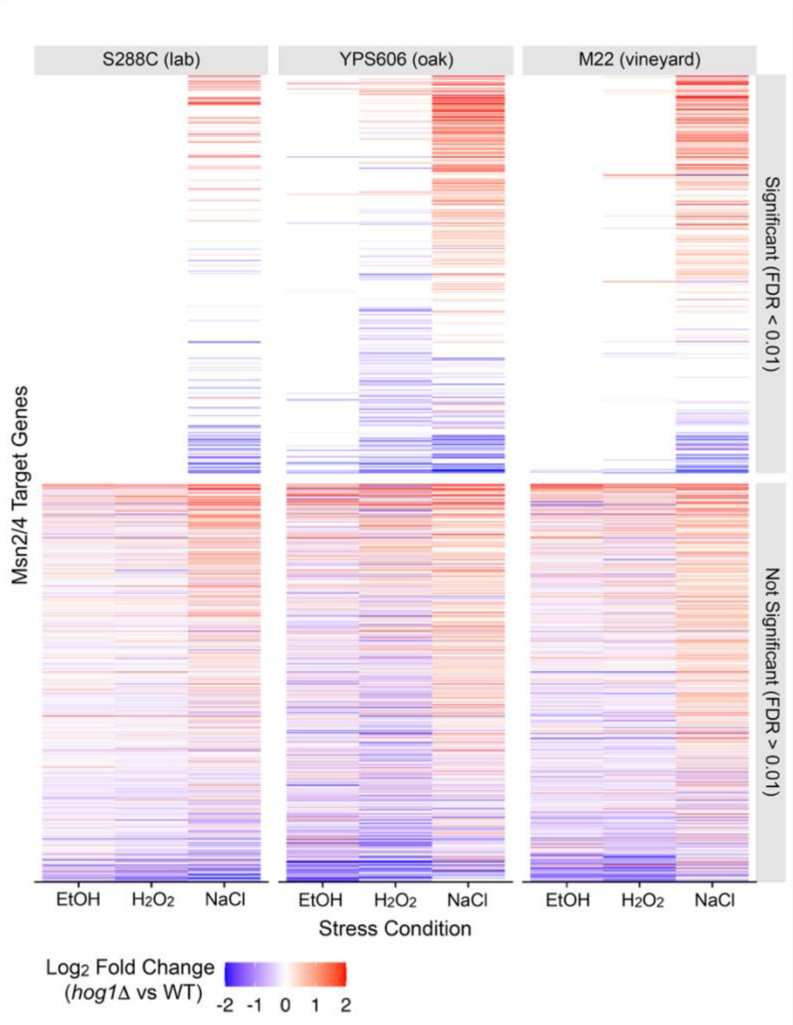

Figure S8. Expression patterns for Msn2/4 targets differ by stress. The heatmap depicts log_2 fold changes in the stress response of *hog1*Δ vs WT strains in each stress condition for known Msn2/4 target genes (from the TFLink Database). The top panel depicts targets that were differentially expressed in at least one strain in the *hog1*Δ vs WT stress response comparison, while the bottom panel depicts genes that were not significantly differentially expressed.

bioRxiv preprint doi: https://doi.org/10.64898/2025.12.28.696518; this version posted December 28, 2025. The copyright holder for this preprint (which was not certified by peer review) is the author/funder, who has granted bioRxiv a license to display the preprint in perpetuity. It is made available under aCC-BY 4.0 International license.

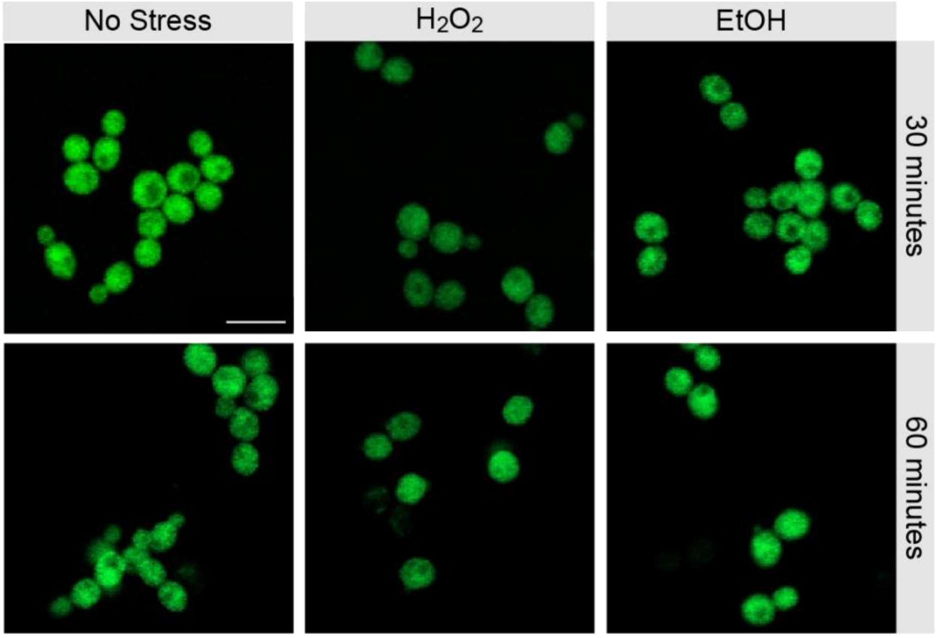

Figure S9. Hog1-GFP remains cytoplasmic during H_2O_2 and ethanol stress at 30 and 60 minutes in YPS606. Each panel depicts live-cell imaging of Hog1-GFP following 30 or 60 min of stress exposure. Scale bar: 10 microns.
